# Characterizing the Chemical Structure of Ti_3_C_2_T_x_ MXene by Angle-Resolved XPS Combined with Argon Ion Etching

**DOI:** 10.3390/ma15010307

**Published:** 2022-01-02

**Authors:** Yangfan Lu, Dongsheng Li, Fu Liu

**Affiliations:** 1 School of Materials Science and Engineering, Zhejiang University, Hangzhou 310027, China; liufu@zju.edu.cn; 2 College of Mechanical Engineering, Zhejiang University, Hangzhou 310027, China; 11825041@zju.edu.cn

**Keywords:** MXene, Ti_3_C_2_T_x_, XPS, angle-resolved, ion etching

## Abstract

Angle-resolved XPS combined with argon ion etching was used to characterize the surface functional groups and the chemical structure of Ti_3_C_2_T_x_ MXene. Survey scanning obtained on the sample surface showed that the sample mainly contains C, O, Ti and F elements, and a little Al element. Analyzing the angle-resolved narrow scanning of these elements indicated that a layer of C and O atoms was adsorbed on the top surface of the sample, and there were many O or F related Ti bonds except Ti–C bond. XPS results obtained after argon ion etching indicated staggered distribution between C–Ti–C bond and O–Ti–C, F–Ti bond. It is confirmed that Ti atoms and C atoms were at the center layer of Ti_3_C_2_T_x_ MXene, while O atoms and F atoms were located at both the upper and lower surface of Ti_3_C_2_ layer acting as surface functional groups. The surface functional groups on the Ti_3_C_2_ layer were determined to include O^2−^, OH^−^, F^−^ and O^−^–F^−^, among which F atoms could also desorb from Ti_3_C_2_T_x_ MXene easily. The schematic atomic structure of Ti_3_C_2_T_x_ MXene was derived from the analysis of XPS results, being consistent with theoretical chemical structure and other experimental reports. The results showed that angle-resolved XPS combing with argon ion etching is a good way to analysis 2D thin layer materials.

## 1. Introduction

2D-crystal materials with the thickness of only one or several atoms usually show special properties and wide application prospect [1]. Graphene, a most typical 2D-crystal material that is composed of carbon atoms with sp^2^ hybrid orbitals and has hexagonal honeycomb lattice, is regarded as a revolutionary material in the future due to its excellent mechanical, electronic, thermal, and magnetic properties [2]. However, graphene is not the only goal of researchers. Some other 2D materials with special properties have attracted many attentions recently [3,4]. MXenes are a series of new 2D-crystal inorganic materials with structures similar to graphene and were reported first by Yury Gogotsi and Michel W. Barsoum from Drexel University at 2011 [5]. The general chemical formula of MXenes can be expressed as M_n+1_X_n_T_x_, where M refers to transition metals (such as Ti, V, Nb, Ta, Cr and Mo); X refers to C or N; n is generally 1–4; and T_x_ refers to surface groups [6,7]. Though it is only ten years since MXenes were discovered, MXenes have become a hot spot in the field of two-dimensional nano materials. With the advantages of high specific surface area, high conductivity, flexible composition and controllable minimum layer thickness, Mxenes show great potentials in energy storage, adsorption, sensors, conductive fillers and other fields [8,9,10,11,12].

At present, the reported MXenes are usually obtained by etching weak A-site element (such as Al or Si atoms) in MAX phase with HF acid or mixed solution of hydrochloric acid and fluoride [5,6,7,8,9,10,11,12,13,14,15,16,17]. The MAX phase is the general term of a series of ternary layered compounds, with the general formula of M_n+1_–AX_n_, where M represents transition metal elements, A mainly refers to group IV elements, X is carbon or nitrogen; n is generally 1 2, 3 and 4 corresponding to 211, 312, 413 and 414 compounds, respectively. The acid etching process would result in a large number of surface functional groups, leading to excellent chemical reactivity and hydrophilicity [13,14,15,16,17,18,19,20]. However, the existence of surface functional groups and their random arrangement would also make it difficult to directly determine the structure of MXenes.

X-ray photoelectron spectroscopy (XPS) is a spectroscopic method to measure the photoelectron energy distribution caused by irradiating the sample with X-ray, and is widely used in chemistry, materials science and surface science areas. It can not only detect the chemical composition of the sample surface, but also determine the chemical state of each element [21,22,23]. In XPS measurement, the photoelectron used for analysis must reach the surface of the sample without energy decline, so it can only be produced at a surface layer (usually within 10 nm). Therefore, XPS is very suitable to characterize the chemical structure of MXenes or similar 2D-crystal materials.

Moreover, the detection depth of XPS is related to the angle between the detector and the sample surface. According to d = 3λcosθ (where d is the detection depth, λ is the attenuation length, θ is the angle between the detector and the normal of the sample surface), when the detector is perpendicular to the sample surface, the detection depth is the largest (still within 10 nm) [24]. The smaller the angle between the detector and the sample surface, the smaller the detection depth. So, by changing the detection angle, we can obtain the information of element composition and chemical state at different depths of the sample super-surface layer. If we want to obtain the chemical information of deeper position (usually more than 10 nm), ion etching can be adapted to peel off the top layer.

There have been some reports about the theoretical structure of MXenes or experimentally characterizing the atomic structure of MXenes by physical method [1,4,5,7,8,9,10,17,19,21,22]. High resolution transmission electron microscope (HRTEM) was usually used to characterize the atomic structure of MXenes from a certain atomic plane [7,19,25,26,27]. However, it is still hard to differentiate the exact element and atom site of surface functional groups from TEM results yet. While surface chemical analysis method XPS has the advantage on determining the chemical composition and chemical state of surface functional groups. There are also some reports about XPS measurement of all kinds of MXenes, [8,10,12,14,21,22,23,25,27,28] in which XPS measurement was mainly used to verify the top surface chemical composition of MXenes. Based on these reports, this paper focused on measuring the surface functional groups and chemical structure of Ti_3_C_2_T_x_ MXene by angle-resolved XPS combined argon ion etching.

## 2. Experimental

Ti_3_C_2_T_x_ MXene was obtained by etching Ti_3_AlC_2_ MAX using mixed solution of hydrochloric acid and lithium fluoride. The detailed preparation process was as follows. First, mix 2 g lithium fluoride and 40 mL 9M hydrochloric acid in a PTEF beaker, and stir the mixed liquid for 30 min with the rotation rate of 400 rpm. Then add 2 g Ti_3_AlC_2_ MAX slowly. The reaction temperature is 35 ℃, and the reaction time is 24 h. Next, centrifugation at 3500 rpm for 10 min was carried out to obtain the sediments. The sediments were washed with deionized water repeatedly until the pH of the upper liquid after centrifugation is 5. Then, ethanol was added into the reaction sediments. Next, the dispersion was sonicated for 1 h and centrifuged at 10,000 rpm for 10 min. The resulting precipitate was sonicated with deionized water for 20 min. Lastly, the Ti_3_C_2_T_x_ MXene dispersion of Ti_3_C_2_T_x_ MXene was dropped on single-crystal silicon substrate to be used for XPS measurement. The XPS measurement was carried out on ESCALAB 250 Xi (Thermo Fisher Scientific Inc., Waltham, MA USA) with the X-ray source of Al K_α_ (1486.6 eV). During the XPS measurements, a flood gun was applied to compensate the surface charge, and further, the binding energy of C–Ti bond was corrected to 282.0 eV. The angle step used in angle-resolved XPS measurement was 5°. The accuracy of the angle-resolved XPS was about ±0.1°. The base pressure of the analysis chamber is 3 × 10^−9^ mbarr. During argon ion etching, the energy of argon ion was 2000 eV, and the beam current was high, the etching time step was 30 s. The thickness of the Ti_3_C_2_T_x_ MXene is about 240 nm measured by a profilometer (Alpha-Step D-100, KLA-Tencor, Milpitas, CA USA). The surface morphology of the produced Ti_3_C_2_T_x_ MXene was characterized by field emission scanning electron microscope (FE-SEM; SU-8100, Hitachi, Tokyo, Japan). The structural property of the sample was characterized by X-ray diffractometer (MAXima XRD-7000, Shimadzu, Kyoto, Japan).

## 3. Results and Discussion

**Figure 1:**
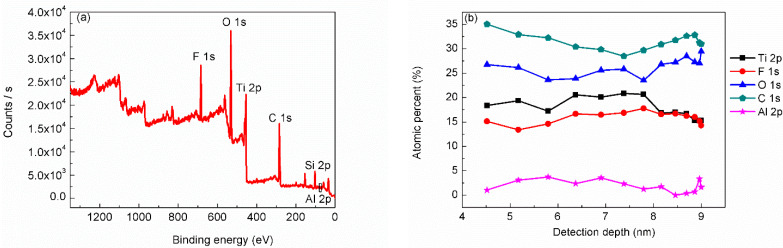
(**a**) Survey scanning obtained on the sample surface at vertical direction, and (**b**) relative ratios of C, O, F, Ti and Al elements obtained at different detection depths.

Before carrying out XPS measurement, the surface morphology and the structural property of the sample were characterized by SEM and XRD, with results as shown in our precious work [29]. The XRD result indicated a sharp peak at 2θ = 6.7° corresponding to Ti_3_C_2_T_x_ MXene and no peaks related to Ti_3_AlC_2_ in MAX phase. That is, the sample was confirmed to be Ti_3_C_2_T_x_ MXene phase with the center-to-center distance of 13.3 Å. Plus, cross-sectional SEM images also indicated the stacked layer structure of the Ti_3_C_2_T_x_ MXene [29]. From the XPS survey scanning (Figure 1a) obtained on the sample surface at the vertical direction, we can find that the sample mainly contains C, O, Ti and F elements. C and Ti should come from the main structure of Ti_3_C_2_T_x_ MXene, and partial C could also come from contamination adsorbed on the sample surface. F should come from the surface group of Ti_3_C_2_T_x_ MXene or the residual reactants. O could be from the adsorbed contamination or the oxidation of the Ti_3_C_2_T_x_ MXene and the Si substrate. Si from the substrate was also detected. Considering the preparation process, Al atoms can also exist in the produced Ti_3_C_2_T_x_ MXene. Though it is hard to distinguish Al element from the survey scanning curve, Al atoms were detected by narrow scanning with very weak signal intensity due to low content, which will be shown later.

Since different detection angle (the angle between the detector and the normal of the sample surface) corresponds to different detection depth. Relative ratios of all the detected elements obtained at different detection angles revealed the composition distribution at different depths, as can be seen from Figure 1b. With increasing of the detection depth, the relative ratio of Ti increased first, and then decreased. What is more, the relative ratio of F showed similar change, with smaller extent. While the relative ratios of C and O showed almost the opposite trend. It can be inferred from these results that there is a layer of C and O atoms adsorbed on the top surface of the Ti_3_C_2_T_x_ MXene. Since the Ti_3_C_2_T_x_ MXene sample was exposed to air before being transferred into the analysis chamber, it is normal that there is an adsorption layer of C and O on the top surface.

**Figure 2:**
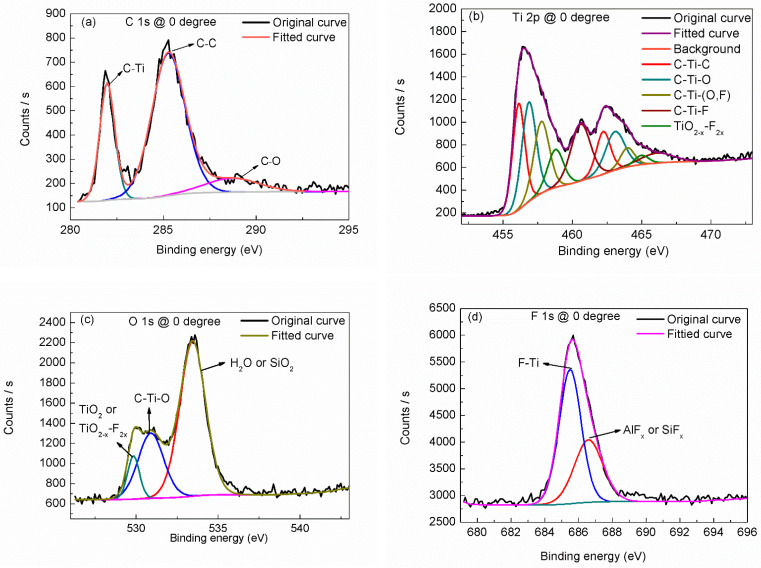
Peak fitting of narrow scanning, (**a**) C1s, (**b**) Ti 2p, (**c**) O 1s and (**d**) F 1s.

Narrow scanning curves of C 1s, Ti 2p, O 1s and F 1s were fitted using surface chemical analysis software “Avantage 5.979”. The peak-fitting results were shown in Figure 2 and Table 1. From the narrow scanning of C 1s, as shown by Figure 2a, we can identify three chemical states. Except the chemical bonds with Ti, there exist adsorbed C–C and C–O bonds, consistent with the above inference. Fitting the Ti 2p narrow scanning (as shown in Figure 2b) shows that there are several kinds of C–Ti–T_x_ bonds (including C–Ti–O, C–Ti–(O,F) and C–Ti–F) except the C–Ti–C bonds in Ti_3_C_2_ compound. There also exists a small amount of TiO_2–x_–F_2x_ bond which may be caused by oxidation [22]. Fitting the O 1s narrow scanning (as shown in Figure 2c) shows that, except bonding to Ti_3_C_2_, some O atoms exist in the form of titanium oxide and more O atoms come from adsorbed H_2_O or SiO_2_. Fitting the F 1s narrow scanning (as shown in Figure 2d) shows that F atoms mainly bond with Ti as surface group or bond with Al as byproduct of the synthesis procedure or bond with Si substrate.

**Figure 3:**
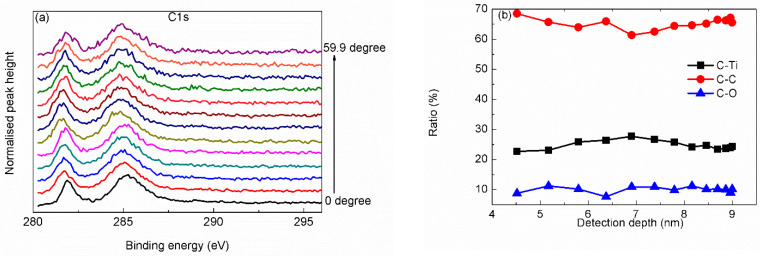

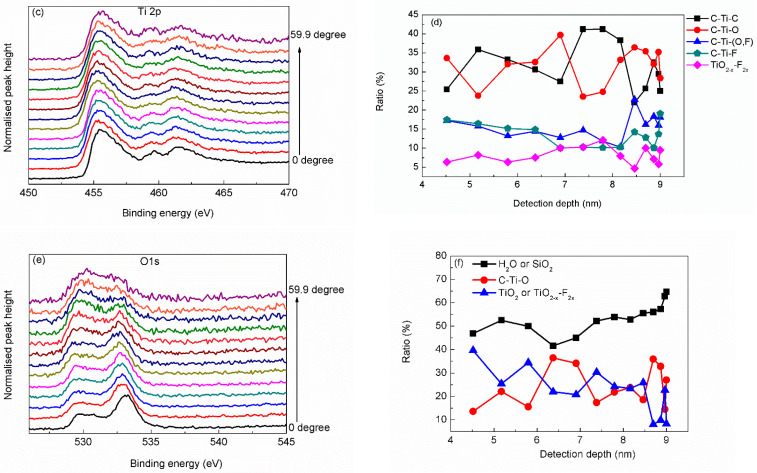
Narrow scanning angle-resolved distribution and state ratio angle-related depth profile of C 1s (**a**,**b**), Ti 2p (**c**,**d**) and O 1s (**e**,**f**).

Figure 3a,c,e show angle-resolved narrow scanning curves of C 1s, Ti 2p and O 1s. Further, ratio depth (within 10 nm) profile of different bonds were obtained from analyzing the angle-resoled narrow scanning curves, as shown in Figure 3b,d,f. According to the ratio depth profile of C–Ti bond (as shown in Figure 3b), the Ti_3_C_2_T_x_ MXene should locate in the subsurface, under the adsorption layer. Ratio depth profiles of different Ti bonds (as shown in Figure 3d) indicate that there may be more than one layer of Ti_3_C_2_, and O/F atoms may exist between the Ti_3_C_2_ layers. Ratio depth profiles of different O bonds (as shown in Figure 3f) approve that O atoms exist not only on the top surface but also with the Ti_3_C_2_ layer. Comparing the ratio depth profiles of C–Ti–C bond and O–Ti–C bond, it is found that there is a staggered distribution between the two bonds, being consistent with the inference that O atoms exist between Ti_3_C_2_ layers acting as surface group. With increasing detection depth, the content of O bonds at 533.4 eV decreases first and increases then. Since the peak at 533.4 eV could be ascribed to either H_2_O or SiO_2_, it is deduced that the peak at 533.4 eV should arise from adsorbed water near top surface and arise from oxide on substrate under the top surface. Therefore, the large amount of adsorbed water near the top surface layer indicates that there could be OH^−^ on the surface of Ti_3_C_2_ layer acting as surface functional group.

**Figure 4:**
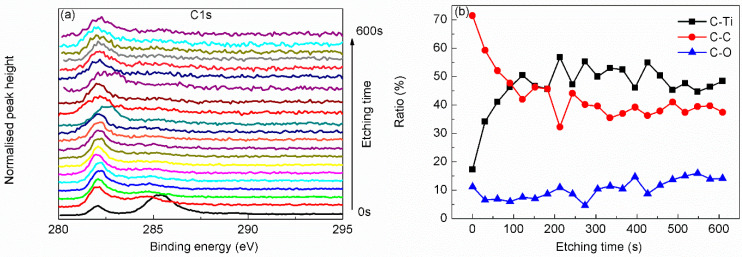
Narrow scanning depth profile (**a**) and state ratio depth profile (**b**) of C 1s.

**Figure 5:**
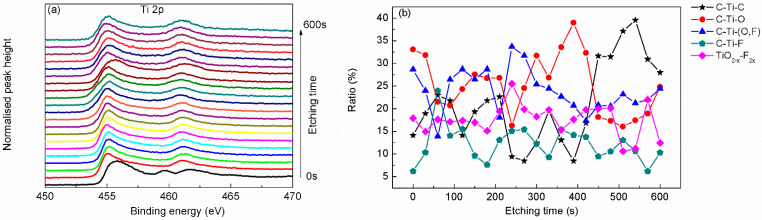
Narrow scanning depth profile (**a**) and state ratio depth profile (**b**) of Ti 2p.

**Figure 6:**
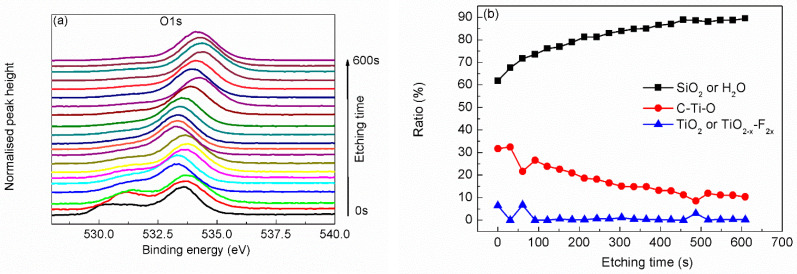
Narrow scanning depth profile (**a**) and state ratio depth profile (**b**) of O 1s.

**Figure 7:**
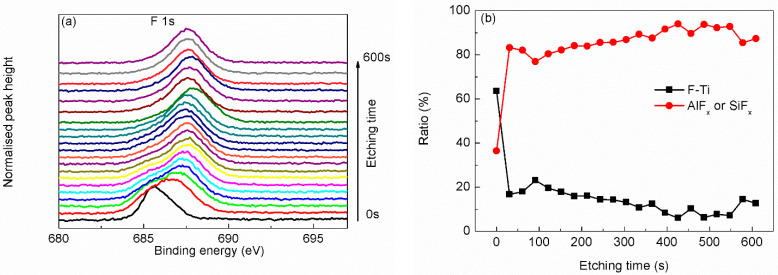
Narrow scanning depth profile (**a**) and state ratio depth profile (**b**) of F 1s.

**Figure 8:**
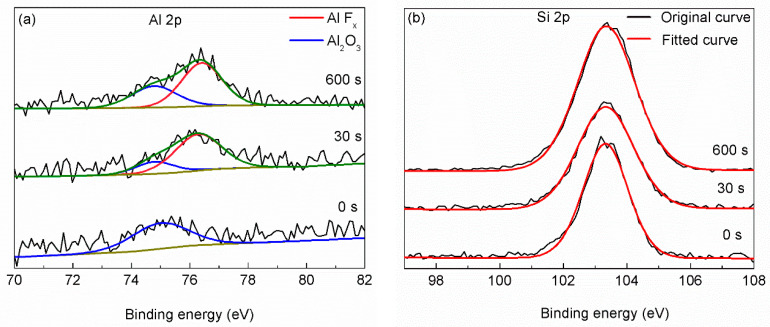
Al 2p (**a**) and Si 2p (**b**) narrow scanning curves obtained before etching and after 30 s and 600 s etching.

In order to further characterize the structure of the Ti_3_C_2_T_x_ MXene, argon ion etching was adopted to analysis the Ti_3_C_2_T_x_ MXene sample layer-by-layer. The top surface of the sample was etched by argon ion, and the newly exposed surface was analyzed by XPS, with the results as shown in Figure 4, Figure 5, Figure 6, Figure 7 and Figure 8.

As seen from the depth profile of C 1s scanning (Figure 4a), the main peak shows indistinct quasi-periodic peak position shifting beneath the top adsorbed layer. As shown in Figure 4b, from the sudden decrease in the proportion of C–C bond below the surface, we can find that the C–C bond mainly comes from the adsorbed contamination. The depth profiles of C–Ti bond and C–O bond show distribution in turns in depth, corresponding to the multi-layer atomic structure of Ti_3_C_2_T_x_ MXene. The generally consistent ratio of C–O bond from the top surface to the inside indicates that O atoms exist through all layers rather than only on the top surface.

Furthermore, the atomic ratio of Ti/C is 3/2 beneath the surface adsorbed contamination layer. Therefore, we can confirm that at the center of Ti_3_C_2_T_x_ MXene there are three layers of Ti atoms and two layers of C atoms stack to each other forming a central Ti_3_C_2_ layer, consistent with the theoretical chemical structure and other TEM experimental reports about the atomic structure of Ti_3_C_2_T_x_ MXene [7,19,25,27].

The depth profile of Ti 2p scanning does not show distinct peak shape changing except that the peak spacing increases, as can be seen from Figure 5a. Peak-fitting ratio depth profiles show that more Ti atoms are bonded with O or F atoms which were called surface groups due to the large specific surface ratio of thin layer structure. As shown by the depth profiles of Ti-related bonds in Figure 5b, O or F atoms related Ti bonds (i.e., C–Ti–O bond, C–Ti–(O, F) bond, C–Ti–F bond and TiO_2–X_–F_2x_ bond) show staggered quasi-periodic distribution related to C–Ti–C bond. It can be concluded that these O or F atoms bonded with Ti atoms should locate at both the upper and lower surface of the Ti_3_C_2_ layer, acting as surface functional groups. This is consistent with the conclusion of C 1s analysis and the theoretical structure of Ti_3_C_2_T_x_ MXene [7,19,25,27]. At the central layer of Ti_3_C_2_T_x_ MXene, Ti atoms bond with C atoms, as seen from the peak sites of Ti–C bond ratio curve in Figure 5b.

Fitted from the O 1s scanning curve (Figure 6a), most detected O atoms are from OH^−^, H_2_O or silicon oxide. What is more, many C–Ti–O bond and TiO_2–X_–F_2x_ bond exist in the layer near the top surface, as shown in Figure 6b. Though the content of C–Ti–O bond decreases with the etching time, the persistent existence of C–Ti–O bond from the top surface to where near the Si substrate shows that O atoms exist at each Ti_3_C_2_T_x_ MXene film. The case of F atoms is similar, as shown in Figure 7. The content of F–Ti bond decreases with etching time to a relative low level. While the content of AlF_x_ or SiF_x_ increases with etching time. The existence of AlF_x_ as byproducts of the synthesis procedure can be verified from narrow scanning of Al 2p as shown in Figure 8a, though the signal of Al 2p scanning is very weak. Since F atoms are not so stable on the F–Ti sites, [22] some F atoms could desorb from Ti_3_C_2_T_x_ MXene and react with Si substrate. SiF_x_ is thought to exist considering the increasing content with etching time and low content of Al atoms, though it is hard to differentiate SiF_x_ and SiO_2_ from Si 2p narrow scanning curves (as seen in Figure 8b).

**Figure 9:**
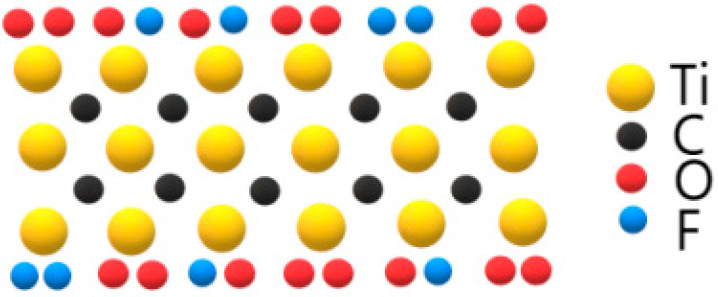
Schematic structure of Ti_3_C_2_T_x_ MXene.

Therefore, based on the above analysis, we can confirm that O atoms, F atoms and hydroxyl are connected to the outer Ti-layer of Ti_3_C_2_ at the upper and lower surface acting as surface functional groups. In other words, the T_x_ in the Ti_3_C_2_T_x_ MXene should include O^2−^, OH^−^, F^−^ or O^−^–F^−^. Since the content of F atoms is much less than O atoms, the ratio of F^−^ functional group should be less than O^2−^ or OH^−^ functional group. Then the sandwich-like atomic structure of the measured Ti_3_C_2_T_x_ MXene can be illustrated as shown by Figure 9. From this perspective, we determined the surface functional groups chemically. That is to say, the chemical structure of Ti_3_C_2_T_x_ MXene can be successfully analyzed by angle-resolved XPS and argon ion etching.

## 4. Conclusions

In conclusion, a Ti_3_C_2_T_x_ MXene sample was analyzed using angle-resolved XPS combined with argon ion etching. The specific surface functional groups were determined to include O^2−^, OH^−^, F^−^ or O^−^–F^−^, and the ratio of O^2−^ or OH^−^ functional group is much higher than that of F^−^ functional group. The chemical structure of Ti_3_C_2_T_x_ MXene were verified to include a central Ti_3_C_2_ layer formed by three layers of Ti atoms and two layers of C atoms stack to each other, and surface functional groups formed by O atoms, F atoms and OH^−^ bonding to Ti_3_C_2_ layer, being consistent with the theoretical structure and other reports. Furthermore, F atoms could desorb from Ti_3_C_2_T_x_ MXene easily and react with Si substrate. From above analysis, angle-resolved XPS combing with argon ion etching is a good way to analysis 2D thin layer materials.

## Figures and Tables

**Table 1:** Peak-fitting results of C 1s, Ti 2p, O 1s and F 1s narrow scanning’s.

Region	BE (eV)	FWHM (eV)	Assignment	References
C 1s	282.0	0.83	C–Ti	[22,23,28]
	285.3	1.77	C–C	[21,22]
	288.7	2.5	C–O	[21,22,23,28]
Ti 2p3 (2p1)	455.1 (461.3)	1.04 (1.23)	C–Ti–C	[23,25]
	456.0 (462.0)	1.22 (1.63)	C–Ti–O	[21,23,28]
	456.9 (463.0)	1.31 (2.29)	C–Ti–(O, F)	[21,23,28]
	457.9 (464.0)	1.67 (2.73)	C–Ti–F	[22,28]
	459.7 (465.2)	1.34 (1.24)	TiO_2–x_–F_2x_	[22,28]
O 1s	529.8	0.89	TiO_2_ or TiO_2–x_–F_2x_	[22,23,25,28]
	530.9	1.9	C–Ti–O	[23,28]
	533.4	1.92	adsorbed H_2_O or SiO_2_	[22,28]
F 1s	685.6	1.67	F–Ti	[21,22,28]
	687.0	1.38	AlF_x_ or SiF_x_	[22]

## Data Availability

The data presented in this study are available on request from the corresponding author.
